# A Comprehensive Approach to Compatibility Testing Using Chromatographic, Thermal and Spectroscopic Techniques: Evaluation of Potential for a Monolayer Fixed-Dose Combination of 6-Mercaptopurine and Folic Acid

**DOI:** 10.3390/ph14030274

**Published:** 2021-03-17

**Authors:** Edvin Brusač, Mario-Livio Jeličić, Matija Cvetnić, Daniela Amidžić Klarić, Biljana Nigović, Ana Mornar

**Affiliations:** 1 Faculty of Pharmacy and Biochemistry, University of Zagreb, A. Kovačića 1, 10000 Zagreb, Croatia; ebrusac@pharma.hr (E.B.); mljelicic@pharma.hr (M.-L.J.); damidzic@pharma.hr (D.A.K.); bnigovic@pharma.hr (B.N.); 2 Faculty of Chemical Engineering and Technology, University of Zagreb, Marulićev trg 19, 10000 Zagreb, Croatia; mcvetnic@fkit.hr

**Keywords:** 6-mercaptopurine, folic acid, compatibility testing, inflammatory bowel disease, forced degradation study

## Abstract

In this work, a systematical compatibility investigation of 6-mercaptopurine and folic acid, two commonly used medications in the treatment of inflammatory bowel disease, for the needs of a fixed-dose combination development strategy is shown. Various techniques and approaches, such as differential scanning calorimetry, isothermal stress testing, attenuated total reflectance–Fourier-transform infrared spectroscopy, dissolution medium stability and forced degradation studies, were used to elucidate the possible interactions from different aspects. The results predominantly point to the absence of physicochemical interactions between the examined substances in a variety of possible conditions. However, the forced degradation of the blend of substances and excipients in basic conditions showed a drastic degradation of 6-mercaptopurine, signifying that attention needs to be directed to the careful selection of the excipients for the formulation. To sum up, our findings indicate that a fixed-dose combination of 6-mercaptopurine and folic acid could be produced using one formulation blend, immensely simplifying its manufacture.

## 1. Introduction

**Figure 1:**
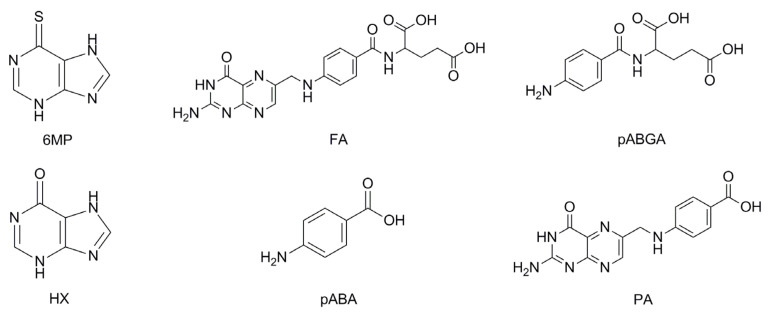
Structures of 6-mercaptopurine (6MP), folic acid (FA) and their selected impurities.

The prevalence of inflammatory bowel disease (IBD), a chronic inflammatory condition affecting not only the gastrointestinal tract but also joints, eyes, liver and skin [1], is on the rise in today’s society, mostly owing to a growing population and increasing life expectancy [2]. A 2017 study estimated about seven million people worldwide to be living with IBD [3]. Although genetic factors play a large role in the pathogenesis, environmental factors such as smoking, diet, occupational lifestyle, gut microbiota and stress can initiate and modulate the disease’s progression [4]. As this is an inflammatory condition mediated by the immune system, anti-inflammatory aminosalicylates, as well as immunosuppressants such as corticosteroids, thiopurines and monoclonal antibodies, are used in therapy. Most often, the small intestine is affected, leading to the malabsorption of many nutrients. Among others, folic acid (FA) deficiency is particularly important, caused by either malabsorption or inadequate dietary intake. Deficiencies of this vitamin are known to primarily cause macrocytic anemia, as well as birth defects in pregnant women, but some studies have also pointed to a possible association between insufficient FA intake and the progression of dementia and Alzheimer’s disease [5], schizophrenia [6] and the progression and invasiveness of gastric cancer [7]. A recent meta-analysis of serum folate and B_12_ levels in IBD patients and healthy controls showed that the patients had significantly lower serum folate levels [8], highlighting the need of FA supplementation in some form. Seeing as the peak age of IBD onset is mid-20s, it is inevitable that the number of drugs the patients have to take will constantly be increasing due to comorbidities or the need for better disease control. This, in turn, leads to lower adherence, most often because of forgetfulness from the sheer number of drugs needing to be administered. Fixed-dose combinations (FDC), also known as “polypills”, often alleviate this obstacle by the administration of two or more active ingredients in one dosage form, improving adherence, health outcomes and quality of life in the treatments of various chronic diseases [9,10,11,12]. As thiopurine immunosuppressants are one of the most prescribed medicines in the treatment of IBD, especially in eliminating corticosteroid dependence and the maintenance of remission when aminosalicylate treatment fails [13], an FDC of an immunosuppressant and FA could offer all of the benefits stated above. However, the chemical and/or physical incompatibilities of the components of the FDC could lead to alterations of the organoleptic properties and stability of the product, as well as formation of unwanted, and possibly pharmacologically active, degradation and interaction products [14]. It follows that the compatibility of the drug substances needs to be investigated first in order to decide on the dosage form and formulation, and therefore, excipients used. If compatibility of the drug substances is proven, a single blend after excipient compatibility testing can be utilized. In the case of incompatibility, technological solutions such as bilayer tablets should be considered. On the other hand, a number of techniques such as thermogravimetric analysis, differential scanning calorimetry (DSC), isothermal stress testing (IST) followed by Fourier-transform infrared spectroscopy (FTIR) or high-performance liquid chromatography (HPLC) assay, X-ray diffraction, dissolution testing and others are used to screen and assess the compatibility of the active substances and planned excipients but, also, of the two or more active substances [15,16,17,18]. Therefore, the aim of this work was to investigate the compatibility of FA and 6-mercaptopurine (6MP) (Figure 1), a drug from the thiopurine class, as a prerequisite for FDC development. The techniques DSC, IST followed by attenuated total reflectance–Fourier-transform infrared spectroscopy (ATR-FTIR) and assays of active substances, dissolution medium stability and forced degradations were applied in order to fortify the conclusions from different aspects. In order to assess the results from IST and forced degradations, a stability-indicating HPLC method was also developed, using some anticipated impurity standards to partially elucidate the degradation pathways. Of the 6MP impurities, hypoxanthine (HX), as the most probable hydrolysis product, was assayed. Of the FA impurities, *p*-aminobenzoylglutamic acid (pABGA), *p*-aminobenzoic acid (pABA) and pteroic acid (PA) were selected as the expected impurities (Figure 1).

## 2. Results and Discussion

### 2.1. Differential Scanning Calorimetry (DSC)

**Figure 2:**
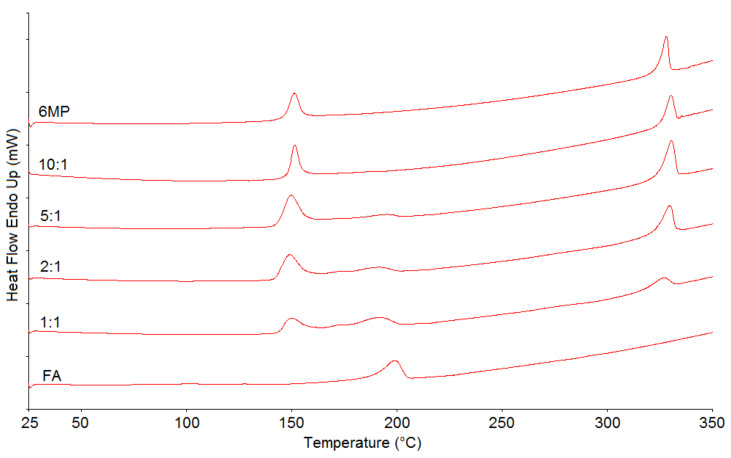
Differential scanning calorimetry (DSC) thermograms of 6MP, FA and their physical blends. Numbers denote 6MP:FA mass ratios in the blends.

Since DSC is a rapid screening technique, it is commonly used in the initial assessment of drug–drug and drug–excipient compatibility [14]. 6MP and FA were thus analyzed separately and in 1:1, 2:1, 5:1 and 10:1 ratios. The results are shown in Figure 2. The thermogram of 6MP showed two distinct and sharp, endothermic peaks at 151.16 and 328.14 °C, with onsets at 146.77 and 323.80 °C, corresponding to the loss of hydrate water and the melting of 6MP, respectively. Similar results have been obtained by other research groups [19,20]. The thermogram of FA showed one relatively broad endothermic peak at 198.86 °C, with an onset at 188.19 °C, possibly corresponding to the melting of the substance. This finding is in accordance with the literature [21]. Regarding the thermograms of the blends, a minor broadening of the peaks can be observed. In addition, the peak temperatures of both 6MP peaks in all the blends shifted slightly either to higher or lower temperatures (shift up to 2.44 °C). As for FA, the shift to a lower temperature was even more evident (up to 7.88 °C). Moreover, the peak of FA in the 5:1 blend was barely visible, even more so in the 10:1 blend. In conclusion, no peaks in the blends disappeared, or new peaks appeared, as compared to pure 6MP and FA, but the peaks somewhat broadened and shifted. This occurrence could simply be due to the mixing of both drugs, lowering the purity of each substance and not their incompatibility [22]. However, the ratio intended for the FDC could not be analyzed using this technique owing to its lower sensitivity, as well as to the intrinsically broad peak of the FA thermal event. Thus, other approaches were considered so as to corroborate the DSC results.

### 2.2. Fourier-Transform Infrared Spectroscopy (FTIR)

**Figure 3:**
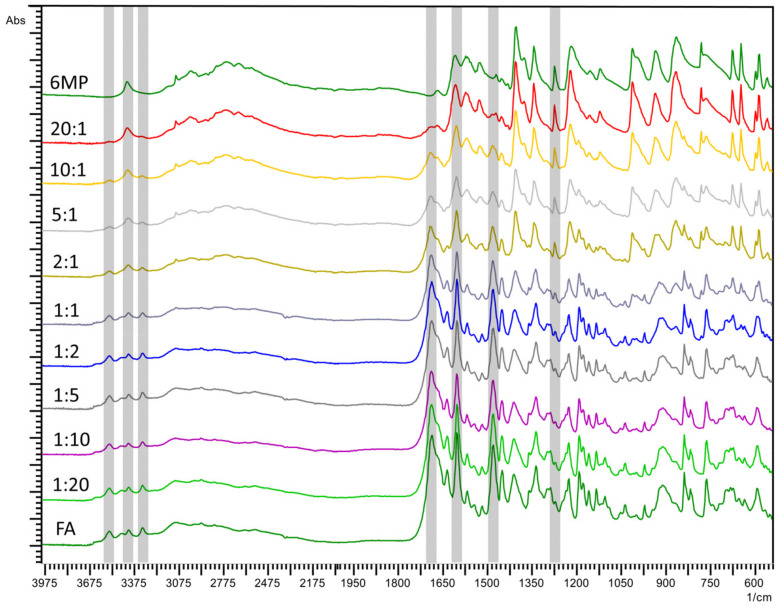
Fourier-transform infrared spectroscopy (FTIR) spectra of 6MP, FA and their physical blends. Numbers denote the 6MP:FA mass ratios in the blends. Greyed areas denote the characteristic bands referred to in the text.

Following DSC experiments, FTIR was utilized to shed light on the possible structural changes. Thermally stressed and nonstressed drug substance and blend samples were first analyzed and compared to detect if there was any effect from thermal stressing. There was no observed disappearance of any bands or the appearance of new ones. The similarity factors (as calculated by IRsolution, correlation coefficients of the regression lines obtained by plotting the absorbances of the two spectra at the same wavenumbers) of the stressed substance or blend spectrum and its nonstressed equivalent were higher than 0.992, implying that heating did not affect the components. Secondly, the characteristic band peaks in the spectra were compared. Figure 3 shows the IR spectra of thermally stressed 6MP, FA and their blends. The FA spectrum shows characteristic bands at 1690, 1603 and 1481 cm^-1^ corresponding to carbonyl stretching, N-H bending of the amide bond and absorption of the phenyl ring, respectively. Additionally, peaks at 3545, 3414 and 3320 cm^−1^ show N-H and O-H stretching. The spectrum of 6MP shows peaks at 3425 and 1275 cm^−1^, corresponding to N-H bending and C=S stretching, respectively. It can be seen that all of these characteristic bands were present in the blends and increased or diminishing proportionally to the ratio of the according substance, indicating that the respective functional groups and structural components remained unchanged.

**Figure 4:**
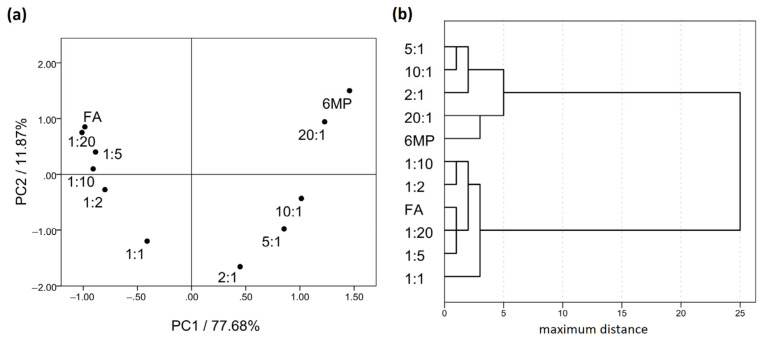
(**a**) Principal component analysis (PCA) scatterplot of 6MP, FA and blends. (**b**) Dendrogram of the cluster analysis (CA) for 6MP, FA and blends. Numbers denote the 6MP:FA mass ratios in the blends.

As overlapping of the bands of each substance can lead to difficulties in data interpretation, a multivariate principal component analysis (PCA) in comparing the spectra was used. Following preprocessing, the data were arranged in a matrix consisting of the substances and their blends as rows and the absorbance values for each spectrum as columns. The region from 550 to 1800 cm^−1^ was chosen, as it contained the majority of the structural information. Upon the data analysis, it was deduced that the first two principal components (PCs), represented by eigenvalues 504.16 and 77.03, together explained 89.55% of the overall variability, which was more than satisfactory, so a scatterplot of PC1 vs. PC2 was chosen for the visualization of the results. The results are shown in Figure 4a, where it is evident that FA is located at the most negative values of the PC1 axis, 6MP on the opposite, most positive values and a trend of increasing the 6MP ratio in the blend along the PC1 axis can be observed. Moreover, the grouping of similar blends can be observed, such as the pure substances and their blends with high ratios. Following PCA, a cluster analysis (CA) was performed, and the resulting dendrogram is shown in Figure 4b. As can be seen, two clusters below the more restrictive Sneath index (33% of the maximum distance) formed. Each cluster consisted of one of the drug substances with its blend of high mass ratios, signifying high similarities between FTIR spectra of the clustered samples. In addition, the subclusters showed pairings of similar ratios, e.g., 6MP and 20:1 blend or FA 1:20 and 1:5 blend, etc. In the case of incompatibility, the PC plot would show no trends along the PC1 axis with the increasing ratios of 6MP. Instead, a separation of 6MP or FA, their positioning in the midst of the PC1 axis or their clustering with the blends predominated by the other substance would likely occur [23]. Such clustering of chemically nonsimilar blends would also be present in the dendrogram. Therefore, it can be concluded that the FTIR spectra of the blends reflect a lack of interaction between 6MP and FA and, consequently, prove their compatibility.

### 2.3. HPLC Method Development

**Figure 5:**
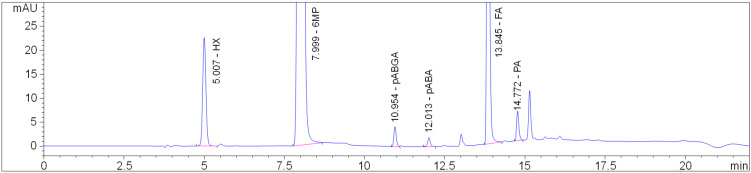
Chromatogram of a standard solution containing 10 µg/mL of hypoxanthine (HX), 500 µg/mL of 6MP, 0.5 µg/mL of p-aminobenzoylglutamic acid (pABGA), 0.125 µg/mL of p-aminobenzoic acid (pABA), 25 µg/mL of FA and 0.5 µg/mL of pteroic acid (PA).

For the IST and stress study compatibility assessments, a HPLC method first needed to be developed. In the preliminary investigations, three column chemistries were evaluated: octylsilyl, octadecylsilyl (both columns 150 × 4.6 mm, 5-µm particle size) and phenyl (150 × 4.6 mm, 2.7-µm particle size). The peak shapes (especially pABGA) were distorted on the C8 and C18 columns, while the phenyl column gave sharp, narrow peaks, favoring good selectivity, so it was chosen for further studies. Ammonium acetate buffers (5 mM) at pH 3.5 and 4.5 as the aqueous mobile phase components were tested but caused lower retention and broader peaks (leading to the increased possibility of coelution in degradations) compared to the chosen aqueous phase consisting of 0.1% (*v*/*v*) formic acid in ultrapure water. Although 6MP and FA are both freely soluble in alkali solutions, the solvent used for standards, blends and placebo dissolution in forced degradation studies should be closer to the neutral pH range. Considering the high amount of 6MP to be dissolved in order to successfully quantify the FA impurities, tetrahydrofuran (THF) was initially examined, owing to good solubility of 6MP in said solvent. However, the degradation of 6MP by 7% was observed after 15 min in 50% (*v*/*v*) THF in H_2_O mixtures, while the principal degradant peak was later observed in oxidative stress, implying the oxidation of 6MP in THF. The use of MeOH/H_2_O mixtures was further tested. Although 6MP showed satisfying solubility (≥1 mg/mL) in pure MeOH, high amounts of the methanolic portion (≥50%) in the solvent caused the HX peak to broaden or not be retained at all. After optimizing the methanolic ratios to find a balance between 6MP solubility and minimizing the injection solvent effects, a mixture of 30% (*v*/*v*) MeOH in H_2_O was used as the sample solvent. A chromatogram of 6MP, FA and selected impurities at their British Pharmacopoeia (BP) limit [24] is shown in Figure 5.

### 2.4. Chromatographic Method Validation

Method validation was carried out according to the ICH guidelines [25] for the selectivity, linearity, precision, accuracy and limits of detection (LOD) and quantification (LOQ) of the impurities (based on three and ten signal-to-noise ratios, respectively) and robustness.

#### 2.4.1. Selectivity

Selectivity was examined on the placebo extracted using the optimized procedure and a standard solution mix spiked in the placebo extract. A visual inspection of the placebo chromatogram (Figure S1, Supplementary Materials) showed no peaks that could affect the analysis. Furthermore, peak purity examination of all analytes determined all the purity factors to be higher than 998.6. The purity of the 6MP and FA peaks was also investigated in forced degradation studies, where the factors were higher than 999.6 for 6MP and 995.4 for FA. Hence, it can be concluded that the developed method was sufficiently selective for the intended purpose.

#### 2.4.2. Linearity and Limits of Detection and Quantitation

The linearity of the drug substances was investigated in the range of 80% to 120% of the nominal values (500 µg/mL for 6MP and 25 µg/mL for FA), while the linearity of the impurities comprised the range from the LOQ to 150% of their BP limit. All linearities were examined on a minimum of five concentrations, and least-squares regression was performed. For the relative response factors (RRF) of the impurities, slopes of the impurity calibration curves were divided with slopes obtained from the curves of active substances diluted to the impurity range. The obtained correlation coefficients were high (above 0.9992), as seen from Table 1. Furthermore, low limits of detection (as low as 0.02 µg/mL) and quantitation (as low as 0.05 µg/mL) were achieved for the impurities. It should be noted that all LOQs were well below the prescribed limits of said impurities in the BP. 

#### 2.4.3. Accuracy and Precision

The samples for accuracy testing were prepared by spiking the analytes in three different concentration levels in the placebo solution, prepared the same as for the sample matrix interference tested in the selectivity study. This method exhibited a more than adequate accuracy, as the recoveries of the drug substances ranged from 99.64% to 100.93%, while those of the impurities spanned from 97.09% to 105.85% (Table 2).

The precision of the chromatographic system was firstly investigated by injecting a working solution (standards dissolved in a placebo solution) nine consecutive times. The relative standard deviations (RSD) of the retention times (lower than 0.12%) and peak areas (lower than 0.32% and 2.60% for the actives and impurities, respectively) imply a stable system suitable for the intended analyses. Afterwards, the precision of the method was examined as intra-day precision (six individually prepared working solutions in the same day) and inter-day precision (three prepared working solutions over the course of three consecutive days). The results (Table 2), expressed as RSD, show very good intra-day precision (lower than 0.49% and 4.34% for the actives and impurities, respectively), as well as inter-day precision (lower than 0.70% and 4.72% for the actives and impurities, respectively).

#### 2.4.4. Robustness

The robustness was examined on the working solution mix by deliberately making minor significant changes in the mobile phase flow (±0.02 mL/min), column temperature (±2 °C) and gradient program (±1%). The RSD of the peak areas was lower than 6.35%, while the RSD of the retention times was lower than 3.93%, showing excellent robustness of the chromatographic procedure. Furthermore, the resolution between the peaks was higher than 2.48 in all cases, showing that minor changes do not affect the separation.

### 2.5. Isothermal Stress Studies

As previously mentioned, isothermal stress testing is often used in assessing the compatibility of drug substances with other substances and excipients potentially used in the formulation [16,18]. When coupled with a technique such as chromatography, its advantages are its high accuracy and precision, as well as ability to detect and identify possible interaction products present in small amounts. For the above reasons, isothermal stress (50 °C for four weeks) was applied to the samples. In addition to drug substance blends and formulation blends being subjected to stress, pure drug substances were also stressed in order to exclude the possible thermal degradation of the individual components. Firstly, the stressed samples were visually compared to the controls (kept in the dark at room temperature), where no visual changes were noticed. The samples were then analyzed using the developed HPLC method. The resulting chromatograms showed no new peaks or an increase of the present impurities in the stressed samples compared to the controls. As seen in Table 3, the difference between the stressed and nonstressed drug substance samples was less than 1.03%, showing a thermal stability of 6MP and FA, as well as no apparent interaction of the drug substances. Moreover, as there was no observed decrease in the 6MP and FA amounts between the formulation blends, it can be presumed that the excipients in the blend are also compatible with both substances.

### 2.6. Stability in Fasted-State Simulated Intestinal Fluid (FaSSIF)

**Figure 6:**
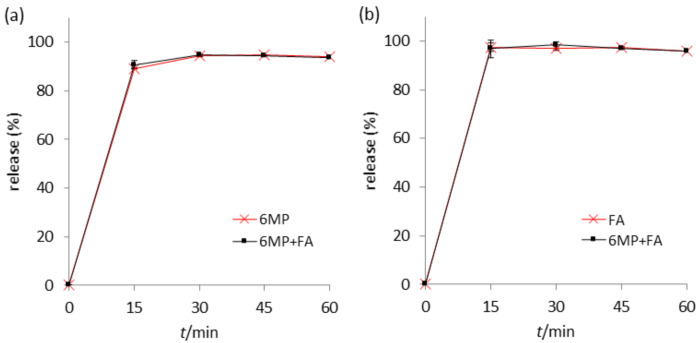
Dissolution profiles of (**a**) MP and (**b**) FA formulations subjected to the dissolution procedure individually and combined. Error bars represent the standard deviation (*n* = 3).

As the conditions, as well as chemical composition, in the human gastrointestinal system are widely different than those during long-term storage, there is a possibility that the active substances could interact with each other. Therefore, the stability of the active substances in the presence of each other in a medium similar to that physiologically present should be assayed. A simulated intestinal fluid mimicking the fasted state was chosen for this purpose, seeing that the compounds are absorbed in the small intestine. Stability testing in a gastric medium was omitted, because the conditions present in the fasted-state stomach are similar to those used in acidic hydrolysis. Moreover, the drugs are not expected to reside in the acidic medium for a longer amount of time, since the fasted-state emptying time for about 85% of the gastric content is half an hour [26]. The testing was conducted on commercially available 6MP and FA tablets individually and in combination in 500 mL of FaSSIF thermostated at 37.0 °C, with the rotation speed of 75 rpm to replicate the physiological conditions as much as possible. Individual testing of 6MP and FA tablets showed the release of active substances after 60 min to be higher than 93.8% (6MP) and 95.6% (FA) of the declared value (Figure 6). When the formulations were subjected to the dissolution medium simultaneously, no remarkable difference was detected in the percentages released at any sampling time point, as the maximum difference in the average drug release in individual and simultaneous dissolutions was lower than 1.5% at all time points for both substances. Furthermore, no additional peaks as compared to individual dissolutions were observed. The RSDs of the same time points in triplicates of all the dissolution tests were lower than 3.6%, indicating formulation homogeneity of the batches. All the above observations point to the absence of chemical interactions of 6MP and FA in FaSSIF, from which a parallel can be drawn to the physiological conditions of the intestinal medium.

### 2.7. Forced Degradation

**Figure 7:**
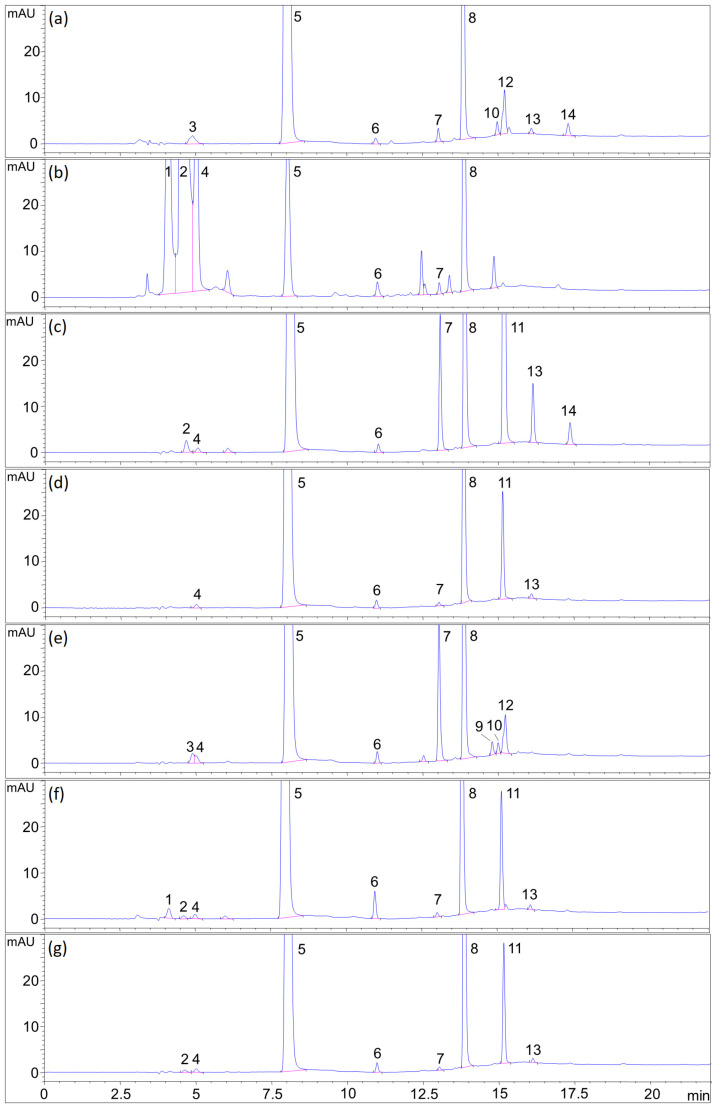
Forced degradation chromatograms of the formulation blend. (**a**) Acidic hydrolysis, (**b**) basic hydrolysis, (**c**) oxidative, (**d**) thermal (solid), (**e**) thermal (solution), (**f**) photolysis (solid) and (**g**) photolysis (solution). Legend: 6MP—5; FA—8; HX—4; pABGA—6; PA—9; 6MP degradants—1, 2, 7, 11, 13 and 14 and FA degradants—3, 10 and 12.

Certain regulatory agencies recommend comparisons of degradation profiles of individual and combined drug substances in order to evaluate their degradability in each other’s presence [27]. To assess the likelihood of 6MP and FA interacting under a variety of possible environmental conditions, studies in acidic, basic, oxidative, thermal and light stress were conducted. The drug substances were stressed individually to gain an insight into their stability, as well as peaks generated. The blends were further stressed in order to evaluate the stability of 6MP and FA in combination with each other and with the excipients. Furthermore, the goal was to ascertain if there are new degradants arising from the latter two sample types that were not observed in the individual drug substances. The placebo and solvent (30% MeOH with stressors, where applicable) were also analyzed to rule out the peaks arising from their degradation. Degradation was carried out either until at least 5% degradation of the more labile active ingredient in one of the sample types or up to a maximum of seven days. For the assessment of compatibility, the degradation profiles and degradation percentages of each sample type under the same stress were evaluated, with the results shown in Figure 7 and Table 4.

#### 2.7.1. Acidic Hydrolysis

Acidic stress was conducted using 0.1 M HCl for four hours at room temperature. FA was shown to be more susceptible to acidic degradation than 6MP. pABGA (marked as 6 in Figure 7) was the only identified impurity arising from the hydrolysis (1.3% compared to the initial concentration of FA), suggesting a cleavage between the pteridine ring and the rest of the molecule. Two more prevalent FA degradants at RRT_FA_ (retention time relative to the retention time of the FA peak) 1.08 and 1.10 were also observed (peaks 10 and 12). In stressing the blends, no additional peaks in the standard or formulation blends emerged, while the losses in analyte content did not differ discernibly.

#### 2.7.2. Basic Hydrolysis

Basic hydrolysis was carried out in 0.1 M NaOH for five days at room temperature. As can be seen from Table 4, the differences in degradation of the individual substances and their blends were lower than 4.6%, while no peaks arose from the interactions. However, in the formulation blend, the peak area of 6MP decreased by 90.0%, as compared to 2.6% in the standard blend, while the decomposition profile of FA remained unchanged. While there were no new peaks, a tremendous increase of peaks of HX (peak 4 in Figure 7, 11.5% compared to the initial 6MP concentration) and degradants at RRT_6MP_ (retention time relative to the retention time of the 6MP peak) 0.51 and 0.56 (marked as 1 and 2) was noticed as compared to the other sample types. Since 6MP only degraded at such a high amount in the formulation blend, and not in the substance blend as well, it can be presumed that one of the excipients present in the blend was responsible for the occurrence. 6MP and FA can be considered intrinsically stable in the basic medium, since the duration of the stress and concentration of the stressor were both relatively high. When inspecting the placebo stress solution chromatogram (Figure S2, Supplementary Materials), multiple peaks could be seen that were not observed in the blank chromatogram or other placebo stressing, which points to chemical changes of excipients in the placebo blend due to the basic pH. Therefore, it can be concluded that the decomposition products of the excipients, and not the presence of FA, led to the increased degradation of 6MP.

#### 2.7.3. Oxidative Stress

Oxidation was achieved using 0.1% (*v*/*v*) H_2_O_2_ for 16 h. As can be seen from the peroxide concentration, this is a very mild stress condition. 6MP was found to be more labile in these conditions (degradations ranging from 4.4% to 6.1%), while the degradant peaks at RRT_6MP_ 1.61, 1.87 and 1.99 (marked as 7, 11 and 13, respectively) increased strikingly, most likely corresponding to some form of dimerization and further oxidation of two 6MP molecules. The FA degradation profile (loss of content up to 1.7%) showed an increase in the pABGA concentration. No interaction peaks were observed.

#### 2.7.4. Thermal Stress

Thermal stressing was done at 60 °C for seven days for solids and five days for solutions. The solid samples were seemingly stable at these conditions (6MP degradation from 0.0% to 1.2%; FA showed no degradation in any of the samples). As for solution stressing, 6MP and FA degraded somewhat evenly (6MP from 1.2% to 4.5% and FA from 4.4% to 7.7% in all samples). The principal impurity of 6MP in this type of stress was peak 7, also present in a large amount during oxidative stress. FA degraded primarily to pABGA (1.1% of the initial FA concentration) and impurities 10 and 12, similar to acidic hydrolysis but, also, PA (numbered 9 in Figure 7e), 1.1% of the initial FA concentration), implying that prolonged exposure to heat leads to the hydrolysis of the peptide bond between pteroic acid and glutamate in FA. Similar to the above degradations, no interaction peaks were found during thermal stress.

#### 2.7.5. Photolysis

For the photolytic studies, the samples were exposed to indirect sunlight for seven days for solids and 15 min for solutions. The exposure of solids to light led to the degradation of FA (2.1% to 6.9%), while 6MP showed superior stability during the course of seven days (no degradation observed). As for the solutions, the exposure of even one hour of FA solution to indirect sunlight caused all the FA to decompose. After exposure of 15 min, the FA content diminished by a small amount (1.4% to 5.1%), while that of 6MP dropped inappreciably (degradation up to 0.5%). This problem can easily be circumvented by using packaging through which light does not penetrate, such as aluminum blisters. In both solid and solution samples, the principal impurity of FA observed was pABGA (4.7% and 1.3% of the initial FA concentration in solids and solutions, respectively), which is in accordance with the literature [28,29,30]. As before, no new peaks arose in the substance and formulation blends.

#### 2.7.6. Summarized Results of Forced Degradation

As can be seen, 6MP was particularly sensitive to oxidation, degrading in even 0.1% H_2_O_2_. On the other hand, FA was susceptible to acidic hydrolysis and photolytic degradation in solids and solutions, while both were equally labile in heated solutions. Thermal stress on solids, as well as basic hydrolysis, did not cause extraordinary degradation. Of the known impurities, pABGA was present in the majority of the stressed samples, followed by HX, while PA was found in the solution thermal stress. pABA was not observed in any of the tests. The degradation profiles of 6MP and FA did not differ substantially in all stressed conditions for the drug substance blends, pointing to possible physicochemical compatibility. During basic stress in the formulation blends, however, 6MP degraded majorly, suggesting an interaction between 6MP and one of the excipients or its degradation products under high pH conditions. The interaction of the excipients with the active substances, particularly in basic conditions, will be further studied during formulation development.

## 3. Materials and Methods

### 3.1. Chemicals and Reagents

As the drug and impurity standards, 6MP (as a monohydrate, >98%, TCI, Tokyo, Japan), FA (USP reference standard, Sigma-Aldrich, St. Louis, MO, USA), HX (≥99%, Sigma-Aldrich), pABA (≥98%, Fluka, Buchs, Switzerland), pABGA and PA (European Directorate for the Quality of Medicines, Strasbourg, France) were used. Folacin^®^ tablets (containing 5 mg of FA, lot no. 115 12718, JGL, Rijeka, Croatia) and Puri-Nethol^®^ tablets (containing 50 mg of 6MP, lot no. 611436, Aspen, Dublin, Ireland) were utilized for formulation blend and dissolution medium stability purposes. Sodium dodecyl sulfate (tested according to National Formulary), sodium hydroxide pellets (ACS reagent, 97.0%), lecithin (from soybeans, ≥99%) and sodium chloride (for analysis) were supplied by Sigma-Aldrich. Methanol (MeOH), gradient HPLC grade, was provided by VWR International (Fontenay-sus-Bois, France), HPLC grade formic acid by Merck (Darmstadt, Germany), hydrochloric acid (37%, for analysis) by Carlo Erba (Val-de-Reuil, France), hydrogen peroxide (30%, p.a.) by T.T.T. (Sveta Nedelja, Croatia) and sodium dihydrogen phosphate dihydrate by Kemika (Zagreb, Croatia). Ultrapure water was produced using an Ultra Clear UV water purifying system (SG Water, Barsbuttel, Germany); resistivity MΩ/cm > 18 at 25 °C and total organic carbon (TOC) < 5 ppb. For the placebo mixture, lactose monohydrate, stearic acid, corn starch (Kemig, Zagreb, Croatia), magnesium stearate (Acros Organics, Princeton, NJ, USA), crospovidone, povidone (Ashland, Rotterdam, The Netherlands) and microcrystalline cellulose (Fagron, Donja Zelina, Croatia) were used.

### 3.2. Sample Preparation

#### 3.2.1. Preparation of Standard Blends, Formulation Blends and Placebo

Standard blends (combined in a therapeutic dose ratio) were prepared by homogenizing 200 mg of 6MP and 10 mg of FA (weighed using a MX5 Microbalance, Mettler-Toledo, Greifensee, Switzerland). Formulation blends were prepared similarly by crushing ten Puri-Nethol^®^ and five Folacin^®^ tablets and homogenizing the mixture. Placebo, i.e., a blend of excipients stated in Section 3.1 mixed in a most common ratio used in tablets [31], was also prepared by homogenizing the substances. All procedures were done using a ceramic mortar and pestle until a visually homogenous blend was achieved.

#### 3.2.2. Preparation of Standard, Placebo and Blend Solutions

Stock solutions of 6MP and FA were prepared in concentrations of 600 and 30 µg/mL, respectively, by adding 30 mg of 6MP, 1.5 mg of FA, 40 mg of placebo and 15.0 mL of MeOH in a 50.0-mL volumetric flask and sonicating for 15 min at 50 °C in an Elmasonic XtraTT ultrasonic bath (Biosan, Riga, Latvia). After cooling to room temperature, ultrapure water was added to yield a solvent concentration of 30% (*v*/*v*) MeOH in water; after which, it was sonicated for an additional 15 min at 50 °C. A stock solution of HX in a concentration of 100 µg/mL was prepared by sonicating the impurity in 20 mM NaOH for 10 min. Stock solutions of pABGA, pABA and PA in concentrations of 10 µg/mL were prepared by dissolving the impurities in 20 mM NaOH and then diluting the solutions tenfold. Working solutions were prepared by appropriately diluting the stock solutions with the placebo extract, prepared in the same manner as the stock solutions but with the omission of drug substances. Stock and working solutions were prepared fresh daily.

Preparation of standard and blend solutions for forced degradation and isothermal stress testing studies was as follows: an accurate amount of 6MP drug substance, FA drug substance, their blend, formulation blend or placebo was dissolved/suspended as described above. For isothermal stress testing and forced degradation study, sample solutions containing the analytes were prepared in concentrations of 500 μg/mL 6MP and 25 μg/mL FA. Acid, base and oxidative degradation stressors were added with the aqueous portion of the solvent. Solution stress was carried out utilizing acid hydrolysis (0.1 M HCl, 4 h at room temperature), base hydrolysis (0.1 M NaOH, 5 days at room temperature) and oxidation (0.1% (*v*/*v*) H_2_O_2_, 16 h at room temperature); thermal degradation (60 °C, 5 days) and photolysis (indirect sunlight, 15 min). Stress on the solids (spread in a thin layer in a Petri dish) was conducted thermally (60 °C, 7 days) and photolytically (indirect sunlight, 7 days). Suspensions were centrifuged at 6000 rpm for 5 min (using a mini G centrifuge, IKA, Staufen im Breisgau, Germany) and injected into the chromatographic system, while the solutions were directly analyzed, omitting this step.

### 3.3. Differential Scanning Calorimetry

DSC measurements were done on pure drug substances and their blends in 1:1, 2:1, 5:1 and 10:1 6MP:FA mass ratios. A Pyris Diamond Differential Scanning Calorimeter (PerkinElmer Inc., Waltham, MA, USA) calibrated with indium (99.98% purity, melting point 156.61 °C and fusion enthalpy of 28.71 J/g) was used. The samples were equilibrated for 2 min at 25 °C and heated to 350 °C by a rate of 10 °C/min under a nitrogen flow of 25 mL/min.

### 3.4. Isothermal Stress Testing

Isothermal stress was applied on pure 6MP and FA substances, their blend and formulation blend. All solid substances were weighed and kept at 50 °C in a thermostated incubator (ES-20/60 Orbital Shaker-Incubator, Biosan, Riga, Latvia) for a period of 4 weeks. A control portion of all samples was kept at room temperature and stored in the dark. After the duration of the period, all examined samples were firstly inspected visually for any changes. For ATR-FTIR measurements, heated drug substances and their blends were used without any further processing. For the assay, the samples were dissolved according to the procedure mentioned in Section 3.2.2 and analyzed using the HPLC method mentioned in Section 3.6. The procedure was conducted in triplicate for every stressed and nonstressed sample.

### 3.5. Attenuated Total Reflectance-Fourier Transform Infrared Spectroscopy

#### 3.5.1. Acquisition of FTIR Spectra

FTIR measurements were conducted on a FTIR-8400S (Shimadzu, Kyoto, Japan) equipped with a PIKE MIRacle ATR sampling accessory (PIKE Technologies, Fitchburg, WI, USA), operated using IRsolution software ver. 1.10 (Shimadzu, Kyoto, Japan). A small amount of sample (thermally stressed and nonstressed drug substances and their blends in the 6MP:FA mass ratios of 20:1, 10:1, 5:1, 2:1, 1:1, 1:2, 1:5, 1:10 and 1:20) was loaded on the FTIR, and the acquisition was done in the spectral range from 400 to 5000 cm^-1^ with a spectral resolution of 1.929 cm^-1^. A total of 45 scans per sample were done and averaged to obtain the final spectrum. The ambient atmosphere was used as the background, which was scanned between each sample.

#### 3.5.2. Multivariate Analysis of FTIR Spectra

For the multivariate analysis of the FTIR results, PCA and CA were used, utilizing SPSS Statistical software ver. 23.0 (IBM, Armonk, NY, USA). Prior to the statistical analysis, a 11 × 649 (rows × columns) matrix of the data was constructed, where the rows represented a substance or blend while the columns represented FTIR absorbance values collected in the range from 550 to 1800 cm^-1^. The data were preprocessed by the standard normal variate method before the PCA and CA [32]. For the CA, Ward’s method of clustering and Euclidean distance between samples were applied.

### 3.6. Chromatographic Conditions

Chromatographic separation was undertaken on an Agilent 1100 Series HPLC (Agilent Technologies, Santa Clara, CA, USA). A CORTECS Phenyl column (4.6 × 150 mm, 2.7-µm particle size) equipped with a CORTECS Phenyl VanGuard cartridge (3.9 × 5 mm, 2.7-µm particle size), both purchased from Waters (Milford, MA, USA), served as the stationary phase. For the elution, 0.1% (*v*/*v*) formic acid in ultrapure water as mobile phase A and 0.1% (*v*/*v*) formic acid in MeOH as mobile phase B were used in a gradient program at a flow 0.4 mL/min as follows: 0–3 min 5% B, 3–10 min 5–60% B, 10–18 min 60% B, 18–20 min 60–100% B and 20–22 min 100% B. The total run time was 30 min to re-equilibrate the column to the initial conditions. Column temperature was set to 30.0 °C, while the injection volume was 5.0 µL for the analyte assay and FA peak purity measurements and 1.0 µL for the 6MP peak purity measurements. After each injection, a needle wash using 50% (*v*/*v*) MeOH in water was used. Detection was carried out using a diode array detector set to 275 nm (bandwidth 4 nm), with 400 nm as the reference wavelength. Peak purity studies were done on all datapoints of the peak, while the similarity factor threshold was 995.

### 3.7. Dissolution Medium Stability

The dissolution stability was investigated in FaSSIF pH 6.5, prepared according to Klein [33] with slight modifications, where sodium dodecyl sulfate in a concentration of 0.1% (*w*/*v*) was used instead of sodium taurocholate. Dissolution was examined in 500 mL of FaSSIF equilibrated at 37.0 °C using a LDLT-A10 dissolution tester (Labtron Equipment Ltd., Fleet, UK) and USP Apparatus 2 setup, while the paddle rotation speed was set to 75 rpm. Dissolution was carried out for 60 min, with sampling intervals every 15 min. Five milliliters of the medium were drawn, filtered and analyzed using a modified previously reported method [34]; after which, the drawn volume was replenished using fresh medium. All tests were done in triplicate.

## 4. Conclusions

In light of all the above results, it can be concluded that 6MP and FA are compatible with each other under a variety of environmental conditions. Although the DSC results were somewhat equivocal and indicative of a potential incompatibility, the FTIR, IST, FaSSIF stability and forced degradation studies all demonstrated no interaction between the substances. This, in turn, signifies no need for a bilayer tablet, facilitating the possible formulation pathway of the potential FDC. The IST studies showed no degradation of the active substances in the formulation blend; however, the stress studies in the basic conditions showed a remarkable decrease in 6MP concentration in the presence of the excipients not observed individually or in a blend with FA. This points to a need for the careful selection of excipients to be used in the final formulation.

## Figures and Tables

**Table 1:** Linearity of the developed chromatographic method. LOD: limit of detection, LOQ: limit of quantitation.

Analyte	Range (µg/mL)	Regression Equation	Correlation Coefficient (*r*)	Relative Retention Time ^1^	Relative Response Factor ^1^	LOD (µg/mL)	LOQ (µg/mL)
6MP	400–600	*y* = 8.48 *x −* 10.37	0.9999	/	/	n/a ^2^	n/a
FA	20–30	*y* = 34.10 *x* + 5.12	0.9999	/	/	n/a	n/a
6MP ^3^	1.00–15.00	*y* = 8.57 *x* − 1.63	0.9997	/	1.00	n/a	n/a
FA ^3^	0.05–0.75	*y* = 33.45 *x* + 0.57	0.9995	/	1.00	n/a	n/a
HX	0.15–15.00	*y* = 12.54 *x −* 0.97	0.9999	0.63	1.46	0.06	0.15
pABGA	0.05–0.75	*y* = 24.95 *x* + 0.04	0.9999	0.79	0.75	0.02	0.05
pABA	0.05–0.20	*y* = 57.51 *x* + 0.25	0.9995	0.87	1.72	0.02	0.05
PA	0.05–0.75	*y* = 40.40 *x −* 0.29	0.9992	1.07	1.21	0.02	0.05

^1^ Expressed in regard to their respective parent drug, ^2^ not applicable and ^3^ ranges used for the impurity relative response factor calculation.

**Table 2:** Accuracy and precision of the developed high-performance liquid chromatography (HPLC) method. RSD: relative standard deviation.

Analyte	Low/Medium/High Concentrations (μg/mL)	Accuracy (Recovery, Mean (%) ± RSD, *n* = 3)			Precision (RSD, %)	
		Low	Medium	High	Intra-Day Precision (*n* = 6)	Inter-Day Precision (*n* = 9)
6MP	400/500/600	99.64 ± 1.73	99.78 ± 0.38	100.09 ± 0.11	0.23	0.28
FA	20/25/30	99.98 ± 1.74	100.13 ± 0.77	100.93 ± 1.06	0.49	0.70
HX	1.50/7.50/12.50	103.82 ± 1.51	100.21 ± 1.72	97.40 ± 0.77	1.64	2.06
pABGA	0.15/0.50/0.75	102.83 ± 4.16	102.15 ± 1.04	101.68 ± 1.03	1.18	3.24
pABA	0.07/0.15/0.20	98.38 ± 3.53	104.63 ± 3.19	105.85 ± 0.48	4.34	4.72
PA	0.15/0.50/0.75	97.09 ± 3.45	104.45 ± 1.23	105.53 ± 1.42	2.23	2.96

**Table 3:** Results of isothermal stress testing at 50 °C for 4 weeks.

Sample		Physical Change (In Comparison To Nonstressed Sample)	Recovery (In Comparison To Nonstressed Sample) Mean (%) ± RSD, *n* = 3
6MP drug substance		no significant changes	100.89 ± 0.44
FA drug substance			99.86 ± 0.75
drug substance blend	6MP		100.37 ± 0.68
	FA		98.97 ± 0.49
formulation blend	6MP		101.09 ± 0.43
	FA		100.94 ± 0.21

**Table 4:** Results of the forced degradation studies.

Stress Type	Degradation Condition	Degradation, %						Degradation Profile Remarks (Peaks Numbered as in Figure 7)
		6MP	FA	Drug Substance Blends		Formulation Blends		
				6MP	FA	6MP	FA	
acid hydrolytic	0.1 M HCl, 4 h	3.1	8.9	n.d. ^1^	10.9	n.d.	9.2	FA degraded to pABGA and impurities 10 and 12
base hydrolytic	0.1 M NaOH, 5 days	7.2	1.8	2.6	1.9	90.0	1.8	6MP markedly degraded in formulation blend
oxidative	0.1% H_2_O_2_, 16 h	4.4	n.d.	6.1	1.5	5.1	1.7	possible dimerization and oxidation of 6MP to degradants 7, 11 and 13
thermal (solid)	60 °C, 7 days	0.7	n.d.	n.d.	n.d.	1.2	n.d.	/
thermal (solution)	60 °C, 5 days	4.5	7.7	1.2	6.2	2.7	4.4	formation of PA
photolytic (solid)	indirect sunlight, 7 days	n.d.	2.1	n.d.	4.4	n.d.	6.9	pABGA as principal FA degradant
photolytic (solution)	indirect sunlight, 15 min	0.5	5.1	n.d.	n.d.	0.2	1.4	pABGA as principal FA degradant

^1^ No degradation observed.

## Data Availability

The data presented in this study are available on request from the corresponding author. The data are not publicly available due to privacy or ethical restrictions.

## Supplementary Materials

The following are available online at https://www.mdpi.com/1424-8247/14/3/274/s1. Figure S1. Chromatogram of placebo solution. Figure S2. Forced degradation chromatograms of placebo. (a) acidic hydrolysis, (b) basic hydrolysis, (c) oxidative, (d) thermal (solid), (e) thermal (solution), (f) photolysis (solid), (g) photolysis (solution).
