# Supplementary figures and images for: A Comprehensive Approach to Compatibility Testing Using Chromatographic, Thermal and Spectroscopic Techniques: Evaluation of Potential for a Monolayer Fixed-Dose Combination of 6-Mercaptopurine and Folic Acid
Source: Pharmaceuticals (Basel). 2021 Mar 17;14(3):274. doi: 10.3390/ph14030274 (PMC8002671; doi:10.3390/ph14030274)

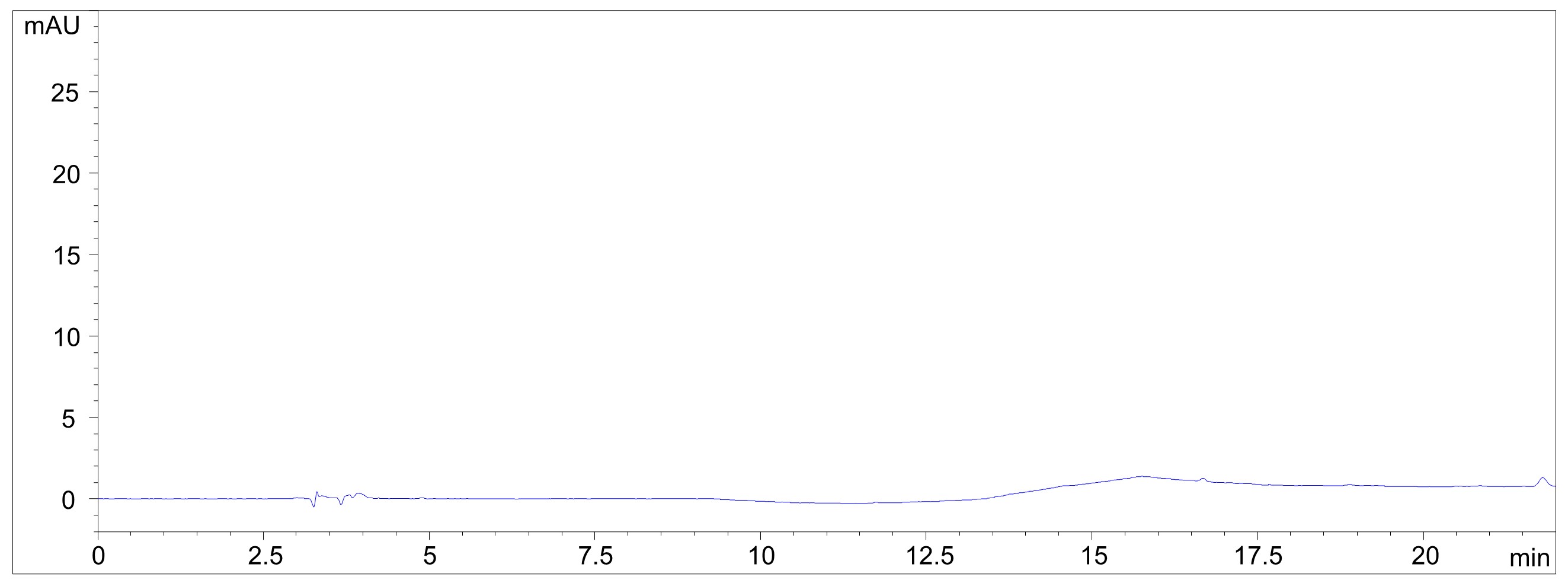

Supplement: Supplementary file 1 [file pharmaceuticals-14-00274-s001.zip › Figure S1, Brusaƒ et al., 2021.jpg]

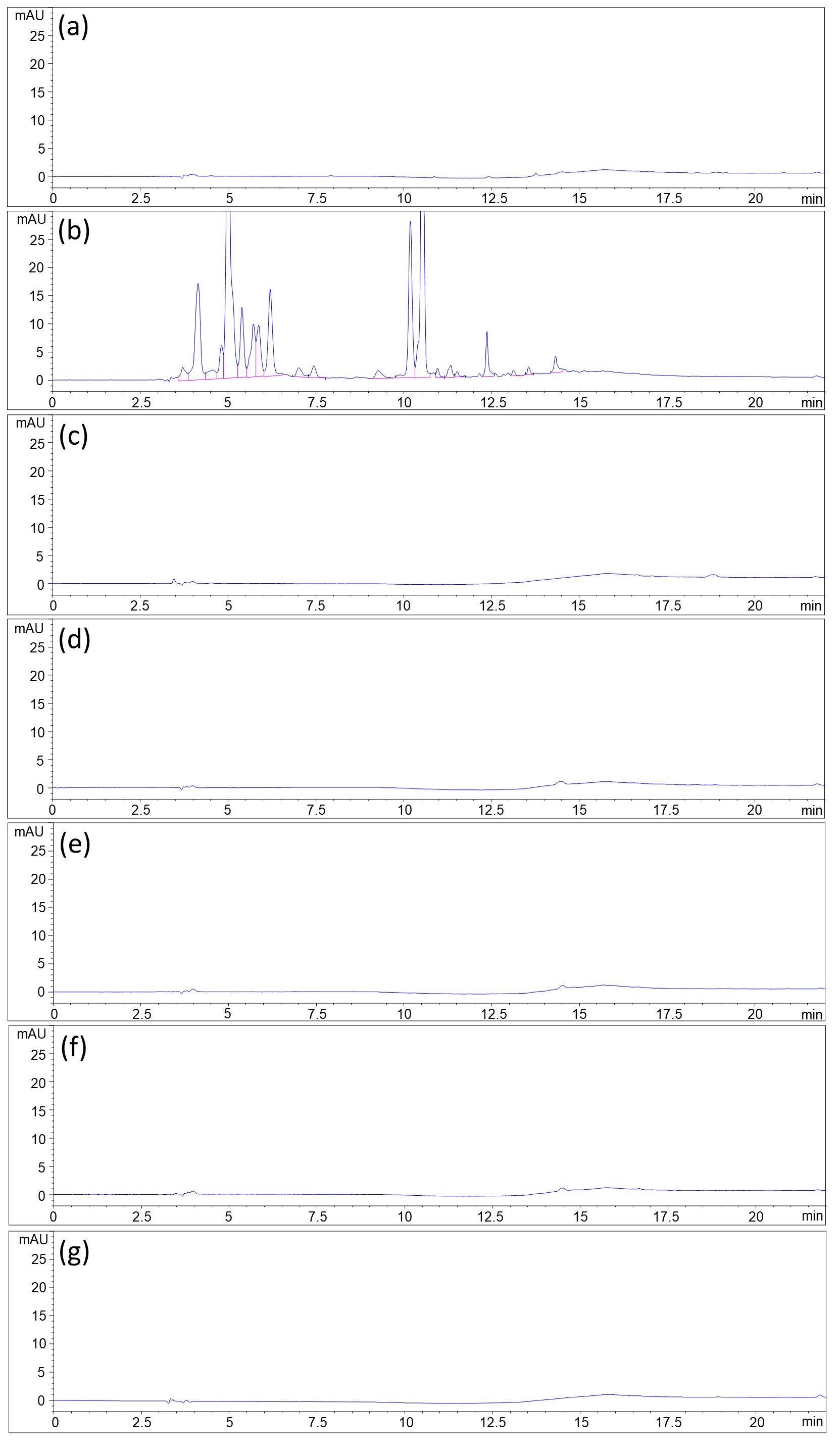

Supplement: Supplementary file 1 [file pharmaceuticals-14-00274-s001.zip › Figure S2, Brusaƒ et al., 2021.jpg]
